# Self-rated health (SRH), recovery from work, fatigue, and insomnia among commercial pilots concerning occupational and non-occupational factors

**DOI:** 10.3389/fpubh.2022.1050776

**Published:** 2023-01-04

**Authors:** Xi Fu, Bingqian Du, Qingsong Chen, Dan Norbäck, Torsten Lindgren, Christer Janson, Roma Runeson-Broberg

**Affiliations:** ^1^School of Public Health, Guangdong Pharmaceutical University, Guangzhou, China; ^2^Guangdong Provincial Engineering Research Center of Public Health Detection and Assessment, Guangdong Pharmaceutical University, Guangzhou, China; ^3^Occupational and Environmental Medicine, Department of Medical Sciences, University Hospital, Uppsala University, Uppsala, Sweden; ^4^Respiratory, Allergy and Sleep Research, Department of Medical Sciences, University Hospital, Uppsala University, Uppsala, Sweden

**Keywords:** recovery from work, demand–control–support, psychosocial work environment, sense of coherence (SOC), type of air plane

## Abstract

**Background:**

This study investigated associations of self-rated health (SRH), recovery from work, fatigue, and insomnia with working conditions, the psychosocial work environment, lifestyle, and sense of coherence (SOC) among commercial pilots.

**Methods:**

A standardized questionnaire was sent to all pilots in an airline company, of whom 354 (61%) responded. Associations were analyzed *via* ordinal and logistic regression with mutual adjustment.

**Results:**

Overall, 21.8% of participants reported low SRH, 13.0% reported not recovering from work during their free time, 61.9% experienced fatigue, and 70.6% experienced insomnia symptoms. A high level of demand at work was associated with poor SRH and more fatigue, and low social support at work was associated with insomnia and poorer recovery from work. Habits surrounding exercise and BMI were associated with SRH. Part-time pilots and captains reported experiencing better recovery from work than their counterparts, while female pilots and younger pilots reported less fatigue. Amount of free time after work and the type of airplane operated were associated with experience of insomnia symptoms. Finally, having a strong sense of coherence was protectively associated with all health outcomes.

**Conclusion:**

The psychosocial environment at work is essential for the health of pilots, and a strong sense of coherence can be protective. Occupational conditions may influence recovery from work, fatigue, and insomnia. Moreover, engaging in exercise, maintaining a healthy weight, working part-time, and having more free time after the working day could improve pilots' health.

## Introduction

The volume of commercial flights rapidly increased between 2001 and 2019, and the annual number of airline flights was 38.9 million in 2019, before the COVID-19 pandemic. Fatigue and sleep problems among pilots are issues that have attracted essential attention due to a reduction in their number of off-duty days and the highly stressful nature of their work. Flight volume fell sharply to 16.9 million in 2020 during the COVID-19 pandemic (1); however, although commercial pilots' working hours were reduced, their fatigue status did not improve. Instead, sleep duration was reported to be shorter, and in-flight sleepiness was reported more frequently during the COVID-19 pandemic compared to the pre-pandemic incidence (2). Due to the increase in competition between airlines, the number of off-duty days was reduced, and work-related stress increased among commercial pilots. At present, flight volume has slightly increased once more, and pilots' health status, fatigue status, and sleep quality continue to be important issues related to flight safety.

Commercial pilots are initially selected partly on the basis of good health, but questionnaire-based surveys have identified a high prevalence among them of various types of medical condition. A recent study of Norwegian commercial pilots found that musculoskeletal complaints (53%) and gastrointestinal problems (60%) were widespread, while allergies, depression, and respiratory symptoms were less common (3). A study of Swedish pilots found that 39.5% had eye symptoms, 39.9% reported nasal symptoms (rhinitis), and 19.8% reported non-specific airway hyperactivity (4, 5). The incidence of doctor-diagnosed asthma among commercial pilots is 2.4 cases per 1,000 person-years, slightly higher than the asthma incidence in the general population (5).

Sleep disturbances are common, especially among pilots operating international flights across time zones (6). A study of commercial pilots in Saudi Arabia found that half of the respondents were at risk of insomnia and fatigue (7). Another survey of international pilots found that the majority of commercial pilots reported fatigue after short-haul (76.5%) as well as long-haul (72%) flights (8). Pilots traveling across different time zones sleep longer after homeward-bound flights than before outward-bound flights (9). They usually recover to baseline with the third recovery sleep (10). Risk factors for fatigue among pilots include long hours of duty, circadian disruptions caused by inter-continental flights, and days of multi-segment duty (11–14). Chronic health problems may influence flight safety, but few pilots admit that they have ever made mistakes during a flight because of fatigue (15).

SRH (self-reported health, also referred to as perceived health) is a widely used indicator of health (16). Measured by a single question, it has been proven to be a reliable predictor of mortality (17) and the development of chronic diseases (18). SRH in the general population is influenced by social differences between countries (19), socioeconomic status (20), and occupational factors (21). We have found no previous study on risk factors for SRH among commercial pilots.

Antonovsky proposed the concept of sense of coherence (SOC). High SOC is a personality trait reflecting the health-promoting capability to cope with stress (22, 23). SOC is a construct consisting of three dimensions, namely comprehensibility, manageability, and meaningfulness, and has been demonstrated to predict various aspects of health (24). The SOC scale has been used in occupational studies (25–27), but we have found no previous research on SOC among commercial pilots.

The psychosocial work environment can influence health and is usually studied under the demand–control–support model (28, 29). Working conditions involving high demands, low control, and low social support are the most harmful (30, 31). Among commercial pilots, a low level of social support has been reported to be associated with sleep problems (32). Furthermore, recovery is an essential psychological process for detachment from work and preparation for new work challenges. Recovery from work can be affected by psychological demands at work, sleep quality, leisure style (33), and vacation time (34, 35). We have found few studies on the associations between psychosocial working conditions and health among commercial pilots (32, 36), and no previous research on recovery from work in this occupational group.

In an investigation of health risks among pilots, it is essential to adopt a holistic perspective, including attention to occupational and non-occupational risk factors. Our hypothesis in this study was that SRH, recovery from work, fatigue, and insomnia among commercial pilots can be influenced by their working conditions (type of aircraft, type of flights, and the psychosocial work environment) as well as their SOC and socio-economic and lifestyle factors. Our first aim was to investigate associations between the psychosocial work environment and SRH, recovery from work, fatigue, and insomnia; our second was to investigate the combined effects of working conditions, SOC, and lifestyle factors.

## Materials and methods

This study formed part of a wider project on working conditions and self-reported health among commercial pilots. A self-administered questionnaire was sent to all Stockholm-based pilots (captains and co-pilots) on duty at a Scandinavian airline company (*N* = 585); 61% of the recipients participated (*N* = 354). The study protocol was approved by the Ethics Committee at Uppsala University, Uppsala, Sweden, and all participants gave their informed consent. Detailed information on the questionnaire has been previously published (32); briefly, it included items on demographic factors, working conditions, the psychosocial work environment, lifestyle and home environment, and SOC. The psychosocial work environment was assessed in accordance with the demand–control–support model by an instrument consisting of 27 questions, which has been validated in a previous study (32). This included five questions on work demands, six on work-related control, and 16 on social support at work. Sense of coherence (SOC) is a salutogenic factor introduced by Antonovsky (22, 23) that reflects an individual's coping abilities. Many instruments have been developed to measure SOC; in the present study, a three-question instrument for measurement of SOC was adapted from Lundberg and Nyström (37). This consisted of one question for each of the three dimensions: (a) manageability: do you usually see a solution to problems and difficulties that others find hopeless? (b) meaningfulness: do you usually feel that your daily life is a source of personal satisfaction? and (c) comprehensibility: do you usually feel that the things that happen to you daily are hard to understand?

### Assessment of the four dependent variables

The questionnaire included one question assessing SRH: “In general, how would you like to describe your health?” (38). This was accompanied by four response options: “excellent,” “very good,” “fair,” or “poor.” A question on recovery from work was adapted from Gustafsson: “Do you feel rested and recovered when you start working again after a couple of days off?” (39). This was accompanied by five response options: “very often,” “quite often,” “sometimes,” “seldom,” or “never.” One question asked about fatigue during work or leisure time. Finally, three questions on sleep disturbances were adapted from a previous sleep questionnaire (40). These questions asked about difficulty in falling asleep, repeated awakenings during sleep, and too early final awakening, with a recall period of 3 months. Four response options were provided for each question: “most of the time,” “sometimes,” “seldom,” or “never.” Both fatigue and insomnia were treated as dichotomous variables, with insomnia being defined as reporting experience of at least one of the three symptoms most of the time or sometimes.

### Statistical methods

Multiple logistic regression was used to analyze associations with fatigue and insomnia (yes/no variables). Ordinal regression was used to examine associations with SRH and recovery from work. For all ordinal regression models, parallel lines were tested to verify that ordinal regression could be used. Initially, health associations were analyzed in models with one exposure factor, adjusting for age and gender (single-factor models). As a next step, mutually adjusted regression models for occupational factors were constructed, including factors with a *p*-value <0.1 from the single-factor analysis, with adjustment for age and gender. The mutually adjusted model with occupational factors was then further adjusted for SOC. Finally, associations identified by the occupational and non-occupational models were selected for final mutual adjustment analysis (inclusion criterion: *p* < 0.1). Pearson's correlation coefficients between the independent variables were calculated. As the correlation between age and year of employment was above 0.7, only age was included in the final mutually adjusted models. All the psychosocial factors were included if any of them met the inclusion criterion of *p* < 0.1. For the logistic and ordinal regression models, odds ratios (*OR*) with a 95% confidence interval (*CI*) were calculated. A *p*-value < 0.05 was considered to be statistically significant. Calculations were carried out using IBM SPSS Statistics 21.

## Results

The majority of respondents (88.9%) were 40–60 years old; 91.0% were men, 5.1% were current smokers, and 22.8% were ex-smokers. A total of 18.2% reported currently using snuff, and 14.8% were ex-users. Almost half were overweight (41.5%), but few (4.1%) were obese (Table 1). Around half (55.3%) had been employed by the same airline company for over 20 years, and 68.9% were full-time employees. Engaging in exercise was very popular, and 71.8% exercised at least twice per week (Table 1).

**Table 1:** Distribution of demographic and occupational variables among commercial pilots (*N* = 354).

**Age**	
31–40	8.8
41–50	60.2
51–60	28.7
61-	2.3
**Gender**	
Man	91.0
Woman	9.0
**Smoking**	
Non-smoker	72.1
Ex-smoker	22.8
Current smoker	5.1
**Oral tobacco (snuff) use**	
Never used	67.0
Have used but quit	14.8
Current snuff user	18.2
**BMI^a^**	
Underweight	0
Normal	54.4
Overweight	41.5
Obese	4.1
**Exercise frequency**	
Sometimes	10.7
Once/week	17.5
2–4 times/week	62.2
>5 times/week	9.6
**Marital status**	
Married/couple	89.4
Weekend couple	3.5
Single	7.1
**Children and age of children**	
None	26.8
7-18 yr	53.1
<6 yr	20.1
**Amount of free time after work**	
Half an hour/day	25.1
1–2 h/day	45.5
3–4 h/day	20.6
5–6 h/day	7.9
**Sleep duration**	
6–8 h	87.6
4–5 h	3.8
>8 h	8.6
**Years of employment^b^**	
5–20	44.7
21–30	36.8
31-50	18.5
**Employment status**	
Full time	68.9
75–80%	28.0
50%	3.1
**Position**	
Captain	61.0
Co-pilot	39.0
**Type of aircraft**	
B737	34.3
MD-80 series	32.0
A330/340	25.8
Saab 2000	7.9

a Body mass index (BMI) was defined as the body mass divided by the square of body height. BMI values were categorized as: underweight (<18.5), normal (18.5–24.99), overweight (≥25.00), or obese (≥30.00). Overweight and obese were merged into a single group (≥25.00) for entry into the statistical models.

b Years of employment is presented here as a summary of the distribution across three ranges, but in the statistical models, it was treated as a continuous variable.

**Figure 1:**
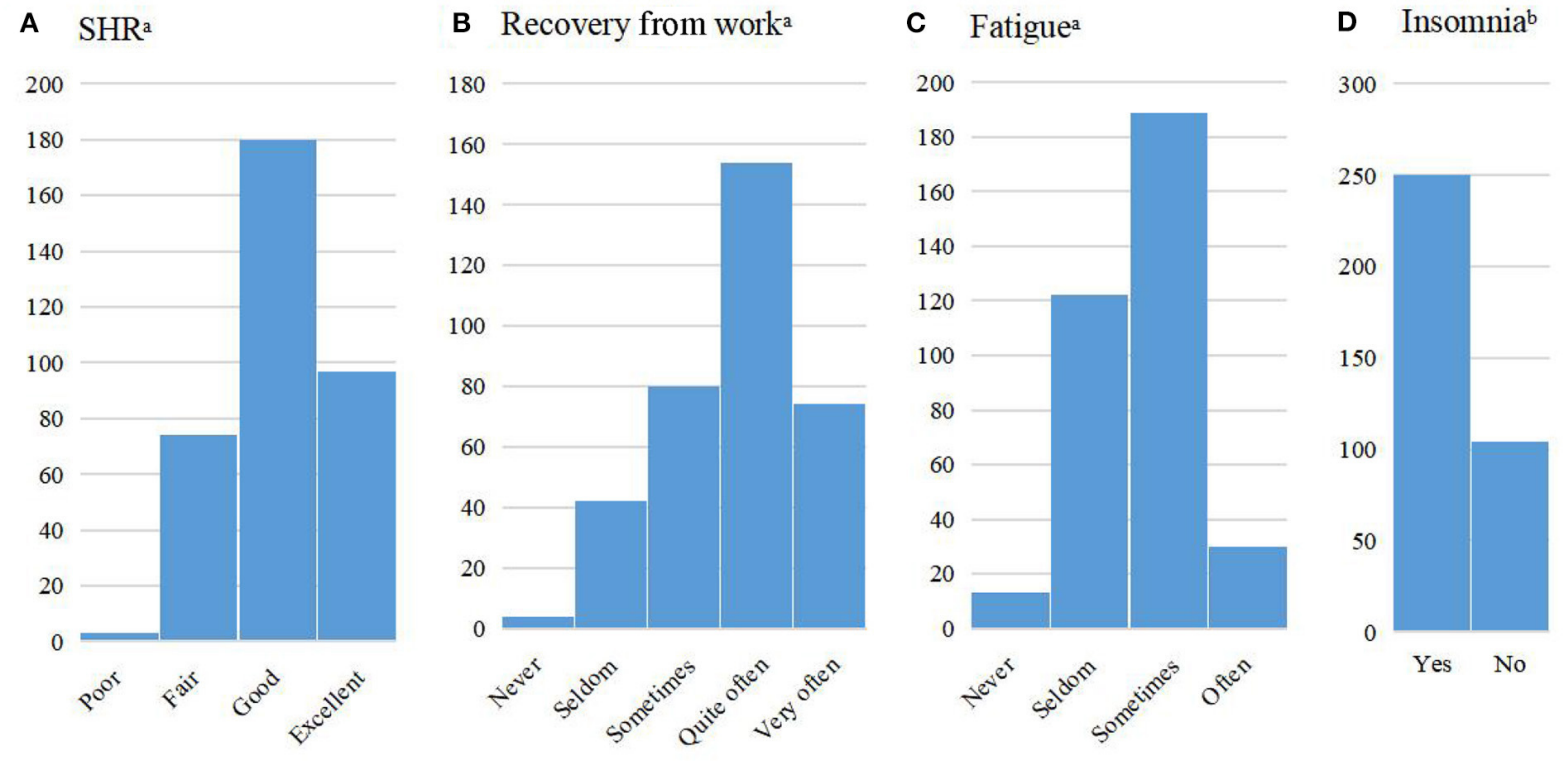
Distribution of responses on self-rated health (SRH), recovery from work, and fatigue, and prevalence of insomnia, among commercial pilots (*N* = 354). **(A)** SHR, **(B)** Recovery from work, **(C)** Fatigue, **(D)** Insomnia. ^a^The scales on which each of these health variables were measured are presented above. Some of the response groups were merged because of small numbers of responses. The categories for each variable entered into the statistical models were as follows: SRH, poor or fair, good, excellent; Recovery, never or seldom, sometimes, quite often, very often; Fatigue, never or seldom, sometimes or often. ^b^The “Yes” category for insomnia represents respondents who reported experiencing any of the following symptoms either sometimes or most of the time during the prior 3 months: difficulty falling asleep, repeated awakenings with difficulty falling back to sleep, or too early final awakening.

A total of 78.2% of the pilots reported good or excellent SRH, and 64.4% reported quite often or very often feeling recovered after several days off work. Fatigue (61.9%) and insomnia (70.6%) were commonly reported (Figure 1). The ranges of scores for work demands (high scores indicating high demand), work control (high scores indicating low control), and social support at work (high scores indicating low support) were 1–15, 3–18, and 0–42, with interquartile ranges of 6–10, 8–11, and 10–19, respectively. The distribution of responses across each dimension of SOC is displayed in Supplementary Table S1 in the Supplementary material. The range of total SOC scores was 1–9, and the interquartile range was 5–6.

Single-factor association analysis was performed for the four dependent variables with respect to each single occupational and non-occupational factor, adjusted for age and gender. Part-time pilots exhibited better recovery (*p* = 0.021). Pilots operating the Saab 2000 aircraft (*p* = 0.041) experienced less fatigue, and those operating the MD-80 series (*p* = 0.012) or Airbus 330/340 (*p* = 0.007) experienced more insomnia, compared to those operating the Boeing 737 (reference). Higher demand and lower levels of social support at work were associated with poorer outcomes on all four dependent variables. A lower level of control at work was also associated with poorer recovery from work. A higher total SOC score was associated with higher SRH (*p* < 0.001), better recovery from work (*p* < 0.001), less fatigue (*p* < 0.001), and less insomnia (*p* < 0.001). Those with a greater sense of manageability, meaningfulness, or comprehensibility reported higher SRH, better recovery from work, less fatigue, and less insomnia. Overweight or obese pilots reported poorer SRH (*p* < 0.001) and more fatigue (*p* = 0.026). Ex-smokers (*p* = 0.003) and current snuff users (*p* = 0.017) reported poorer SRH as compared to non-smokers and non-snuff users, respectively. Pilots in a “weekend couple” relationship reported lower SRH (*p* = 0.037) and poorer recovery from work (*p* = 0.021). Those with pre-school children (aged 0-6 y) at home also reported poorer recovery from work (*p* = 0.009). Pilots who engaged in frequent exercise reported higher SRH (*p* < 0.001) and better recovery from work (*p* = 0.009). Those with more free time after work reported higher SRH (*p* = 0.040), better recovery from work (*p* < 0.001), and less insomnia (*p* = 0.005). Finally, pilots who slept <5 h per night (short sleepers) reported lower SRH (*p* = 0.042) and poorer recovery from work (*p* = 0.014) (Supplementary Table S2).

**Figure 2:**
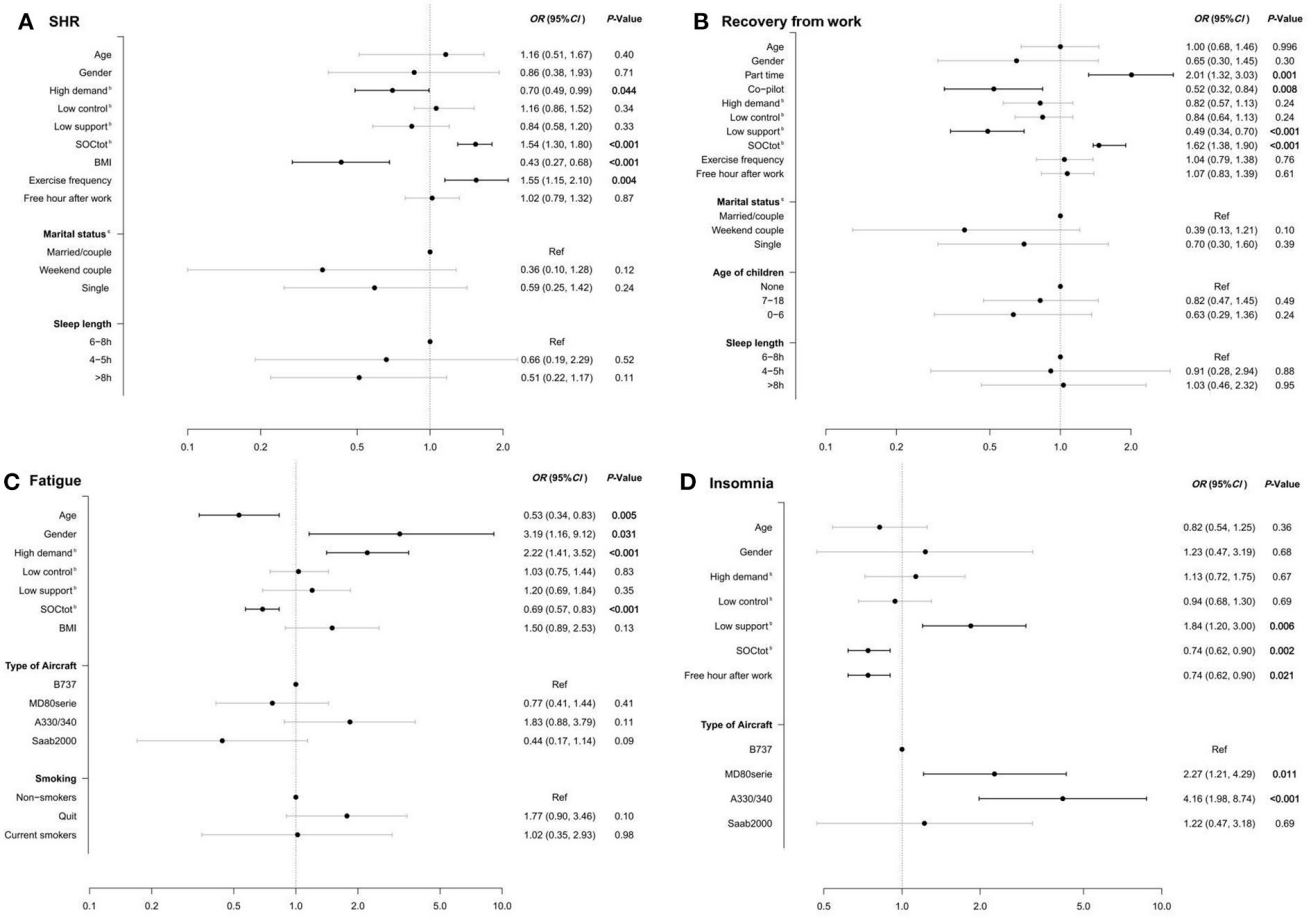
Associations of SRH, recovery from work, fatigue, and insomnia with selected occupational and non-occupational factors in a multiple regression model. **(A)** SHR, **(B)** Recovery from work, **(C)** Fatigue, **(D)** Insomnia. ^a^Associations between dependent variables and the factors included were calculated *via* ordinal regression models, adjusted for age and gender. ^b^Associations of the dependent variables with overall sense of coherence (SOCtot) and with psychosocial variables were calculated on the basis of their interquartile range. ^c^“Couple” was defined as being in a stable relationship in which the respondent lives with their partner. “Weekend couple” was defined as being in a relationship in which the respondent lives separately from their partner, and they usually meet on weekends.

Figure 2 presents the results from the final mutually adjusted models. Associations were identified between aspects of the psychosocial work environment and all health outcomes. Specifically, high work demand was associated with reduced SRH (*p* = 0.044) and increased fatigue (*p* < 0.001), while low social support was associated with poorer recovery from work (*p* < 0.001) and increased insomnia (*p* = 0.006). Type of aircraft was related to fatigue (*p* = 0.032) and insomnia (*p* = 0.003): specifically, pilots operating the MD-80 series aircraft (*OR* = 2.27, 95% *CI*: 1.21–4.29) or Airbus330/340 (*OR* = 4.16, 95% *CI*: 1.98–8.74) had higher rates of insomnia, compared to those operating the Boeing 737 aircraft as a reference. Co-pilots reported experiencing poorer recovery from work than captains (*p* = 0.008), and older pilots reported less fatigue (*p* = 0.005). Part-time work was associated with improved recovery from work (*p* = 0.001). Overweight or obese pilots reported lower SRH (*p* < 0.001), and engaging in exercise more frequently was associated with higher SRH (*p* = 0.004). Finally, more free time after work was associated with reduced insomnia (*p* = 0.021).

## Discussion

In summary, the findings indicated that a poor psychosocial environment at work (i.e., highly demanding work or low levels of control over one's work) can adversely influence pilots' self-rated health, recovery from work, fatigue, and insomnia. Avoiding becoming overweight or obese and engaging in regular physical exercise are important lifestyle factors in promoting health. Furthermore, operating intercontinental flights can increase insomnia among pilots and working part-time can improve their recovery from work. Similarly, having more free time after work can reduce insomnia. Beyond these factors, a strong sense of coherence is also beneficial for all four aspects of health that were examined in this study.

Our study has several strengths. It is a unique study on risk factors and health-promoting factors for self-rated health, recovery from work, fatigue, and insomnia among commercial pilots. The study had a reasonable response rate (61%), and there were no differences in age or gender between participants and non-participants. Thus, selection bias after the point of employment is not likely to have exerted any major influence on our results. However, since pilots are initially selected partly on the basis of being in good health, they are not comparable with the general adult population. An additional strength is that we employed statistical models with mutual adjustment for various occupational and non-occupational factors.

One limitation of the study is that we collected only self-reported data, which could create information bias. However, we observed associations between specific dependent variables and specific risk factors, rather than a general presence of many associations with the same order of magnitude. Thus, it is not likely that information bias underlies the patterns observed. A further limitation is that we recruited pilots only from a single airline, which limits the study's external validity; moreover, the cross-sectional design of the study limits the ability to draw conclusions about causation. Additionally, although we included several factors reflecting working conditions in this study (including position, type of aircraft operated, and full- vs. part-time duty), no environmental measurements were taken in the aircraft. Our previous studies have reported on various microbial exposures in aircraft cabins (41, 42), which have an important impact on human health (43–45). Environmental exposure is difficult to quantify, because aircraft and airlines change every duty day. Further studies are needed to assess the combined impact of working conditions, the psychosocial work environment, and environmental exposure in aircraft.

We observed that SOC was a highly protective factor for pilots' health: specifically, a high SOC was protectively associated with all four health outcomes in single-factor analyses and in the mutually adjusted models. We have found no previous study on SOC among commercial pilots; however, recent studies of workers in other occupations have reported the same protective effect of SOC. A high SOC is associated with better health among Hungarian midwives, and negatively associated with work-related stress in hospitals (46). Additionally, a recent Swedish study has reported that SOC could act as a buffer against work-related stress among teachers (47). SOC represents a salutogenic coping ability that is considered to be relatively stable in adulthood (48, 49), but can nevertheless increase as a result of positive life events (50) or decrease as a result of adverse or drastic life events (51). Several studies have indicated that certain interventions may improve SOC. Such interventions include mindfulness (52), movement, sports and games (53), and activities that facilitate reflection on one's SOC (50). A recent study has reported that resistance training exercise significantly elevates SOC levels among older adults (65–75 years old) (54).

The psychosocial work environment was found to be related to all four health outcomes in single-factor analyses. In the mutually adjusted models, high demand was associated with poor SRH and an elevated incidence of fatigue, and low social support at work was associated with poor recovery from work and an elevated incidence of insomnia. Our results concerning the association between work demands and SRH are consistent with those of several previous large European studies (55, 56). To improve their pilots' psychosocial work environment, airline companies should focus on optimizing social support and, if possible, reducing the demands placed on them at work.

In the following part of the discussion, we focus on factors other than SOC and the psychosocial work environment, discussing associations identified for each health variable. Overall, a total of 21.8% of the pilots reported only poor or fair SRH. We found no previous study on SRH in commercial pilots. Pilots who were overweight or obese had poorer SRH, and those who engaged in frequent physical exercise (at least twice a week) had better SRH. Negative associations between obesity and SRH have been reported previously in large population studies (57, 58). Physical activity has been reported to be associated with SRH in the general population in Seoul, South Korea (59), and in Australia (60).

Our question on recovery from work was adapted from one used with government employees in a previous publication (39). We found that one third (35.6%) of the pilots did not feel recovered after several days off work. Co-pilots reported poorer recovery from work than flight captains, and those working part-time reported better recovery from work. The major differences between captains and co-pilots are rank and salary; captains take responsibility for their flights, and co-pilots take orders from the captain. To our knowledge, our study is the first on recovery from work among commercial pilots.

A majority (61.9%) of the pilots reported experiencing fatigue (sometimes or often). In our previous study, in which we used a different question to measure fatigue, 29.9% of pilots reported weekly fatigue, and 82.8% of pilots reported any experience of fatigue during the prior 3 months (4). Female pilots experienced more fatigue, a finding which is in agreement with that of a previous study reporting a similar gender difference (61). Moreover, older pilots experienced less fatigue, in contrast with the findings of a large population study in the UK reporting that fatigue increases with age (61). However, older pilots were more experienced and their duty schedules were less stringent.

More than two thirds of the pilots (70.6%) reported experiencing insomnia. In addition to the influence of SOC and the psychosocial work environment, being overweight or obese increased insomnia and having more free time after work reduced insomnia. Moreover, the type of aircraft operated also influenced the prevalence of insomnia. The Airbus 330/340 was the only aircraft type used among the respondents for intercontinental flights. As expected, pilots on these intercontinental flights experienced more insomnia than those operating flights within Europe (reference group: Boeing 737 operators). Moreover, pilots who operated MD-80 aircraft experienced more insomnia than those operating the Boeing 737. The Boeing 737 and MD8-0 are both narrow-body aircraft operated over short and medium ranges; thus, it is possible that a factor other than flight duration could be the cause of this difference.

In conclusion, occupational and non-occupational factors have a combined impact on pilots' health. Further studies investigating health associations with exposure to particular environmental factors in either the home or the work environment should use multi-dimensional modeling to assess these associations. For commercial pilots, a positive psychosocial environment is essential for good health. Additionally, a strong sense of coherence can be an important health-promoting personality-related factor in self-rated health, recovery from work, fatigue, and insomnia. To promote good health among commercial pilots, airline companies should invest more resources into improvements to their psychosocial work environment: in particular, they should create the perception of appropriate levels of demand and strong social support at work. Training aiming to improve pilots' SOC is advisable both at the company level and for individuals. Moreover, maintaining a healthy lifestyle is beneficial for health: for instance, avoiding becoming overweight or obese, engaging in regular physical exercise, and maintaining a schedule that includes ample free time after work.

## Data availability statement

The raw data supporting the conclusions of this article will be made available by the authors, without undue reservation.

## Ethics statement

The studies involving human participants were reviewed and approved by the Ethics Committee at Uppsala University, Uppsala, Sweden. The patients/participants provided their written informed consent to participate in this study.

## Author contributions

XF and BD drafted the manuscript and conducted statistical analyses. RR-B and TL were involved in data acquisition, research, and interpretation. DN, CJ, and QC provided a critical review of the manuscript. All authors participated in the conceptualization and implementation of the study, and read and approved the final version of the article.

## References

[1] Mazareanu E. Number of Flights Performed by the Global Airline Industry from 2004 to 2022. (2021). Hamburg, Germany: Statista. Available online at: https://www.statista.com/statistics/564769/airline-industry-number-of-flights/

[2] Hilditch CJ Flynn-Evans EE. Fatigue, Schedules, Sleep, and Sleepiness in U.S Commercial Pilots During COVID-19. Aerosp Med Hum Perform. (2022) 93:433–41. 10.3357/AMHP.6031.2022 35551720

[3] Omholt ML Tveito TH Ihlebæk C. Subjective health complaints, work-related stress and self-efficacy in Norwegian aircrew. Occup Med (Lond). (2017) 67:135–42. 10.1093/occmed/kqw127 27683875

[4] Fu X Lindgren T Norbäck D. Medical symptoms among pilots associated with work and home environments: a 3-year cohort study. Aerosp Med Hum Perform. (2015) 86:458–65. 10.3357/AMHP.4216.2015 25945663

[5] Fu X Lindgren T Wieslander G Janson C Norbäck D. Respiratory illness and allergy related to work and home environment among commercial pilots. PLoS ONE. (2016) 11:e0164954. 10.1371/journal.pone.0164954 27741314 PMC5065138

[6] Graeber RC Lauber JK Connell LJ Gander PH. International aircrew sleep and wakefulness after multiple time zone flights: a cooperative study. Aviat Space Environ Med. (1986) 57:B3–9. 3800828

[7] Alzehairi A Alhejaili F Wali S AlQassas I Balkhyour M Pandi-Perumal SR. Sleep Disorders Among Commercial Airline Pilots. Aerosp Med Hum Perform. (2021) 92:937–44. 10.3357/AMHP.5809.2021 34986931

[8] Venus M Holtforth MG. Short and long haul pilots rosters, stress, sleep problems, fatigue, mental health, and well-being. Aerosp Med Hum Perform. (2021) 92:786–97. 10.3357/AMHP.5812.2021 34641999

[9] Eriksen CA Akerstedt T. Aircrew fatigue in trans-Atlantic morning and evening flights. Chronobiol Int. (2006) 23:843–58. 10.1080/07420520600865574 16887752

[10] Lowden A Akerstedt T. Eastward long distance flights, sleep and wake patterns in air crews in connection with a two-day layover. J Sleep Res. (1999) 8:15–24. 10.1046/j.1365-2869.1999.00129.x 10188132

[11] VAN DEN Berg MJ Signal TL Gander PH. Fatigue risk management for cabin crew: the importance of company support and sufficient rest for work-life balance-a qualitative study. Ind Health. (2020) 58:2–14. 10.2486/indhealth.2018-0233 30996214 PMC6997722

[12] Wen CCY Nicholas CL Clarke-Errey S Howard ME Trinder J Jordan AS. Health Risks and Potential Predictors of Fatigue and Sleepiness in Airline Cabin Crew. Int J Environ Res Public Health. (2020) 18:13. 10.3390/ijerph18010013 33375088 PMC7792809

[13] Goffeng EM Wagstaff A Nordby KC Meland A Goffeng LO Skare Ø. Risk of Fatigue Among Airline Crew During 4 Consecutive Days of Flight Duty. Aerosp Med Hum Perform. (2019) 90:466–74. 10.3357/AMHP.5236.2019 31023407

[14] Honn KA Satterfield BC McCauley P Caldwell JL Van Dongen HP. Fatiguing effect of multiple take-offs and landings in regional airline operations. Accid Anal Prev. (2016) 86:199–208. 10.1016/j.aap.2015.10.005 26590506

[15] Aljurf TM Olaish AH BaHammam AS. Assessment of sleepiness, fatigue, and depression among Gulf Cooperation Council commercial airline pilots. Sleep Breath. (2018) 22:411–9. 10.1007/s11325-017-1565-7 28884322

[16] De Bruin A Picavet HS Nossikov A. Health interview surveys. Towards international harmonization of methods and instruments. WHO Reg Publ Eur Ser. (1996) 58:i–xiii, 1–161. 8857196

[17] Taloyan M Leineweber C Hyde M Westerlund H. Self-rated health amongst male and female employees in Sweden: a nationally representative study. Int Arch Occup Environ Health. (2015) 88:849–59. 10.1007/s00420-014-1014-x 25527963

[18] Latham K Peek CW. Self-rated health and morbidity onset among late midlife US adults. J Gerontol B Psychol Sci Soc Sci. (2013) 68:107–16. 10.1093/geronb/gbs104 23197340 PMC3605944

[19] Schütte S Chastang JF Parent-Thirion A Vermeylen G Niedhammer I. Social differences in self-reported health among men and women in 31 countries in Europe. Scand J Public Health. (2013) 41:51–7. 10.1177/1403494812469854 23341354

[20] Bethune R Absher N Obiagwu M Qarmout T Steeves M Yaghoubi M. Social determinants of self-reported health for Canada's indigenous peoples: a public health approach. Public Health. (2019) 176:172–80. 10.1016/j.puhe.2018.03.007 29666024

[21] Fujishiro K Koessler F. Comparing self-reported and O^*^NET-based assessments of job control as predictors of self-rated health for non-Hispanic whites and racial/ethnic minorities. PLoS ONE. (2020) 15:e0237026. 10.1371/journal.pone.0237026 32760109 PMC7410273

[22] Antonovsky A. Health, Stress, and Coping. New Perspectives on Mental and Physical Well-Being. San Francisco: Jossey-Bass (1979). p. 12–37

[23] Antonovsky A. Unravelling the Mystery of Health. How People Manage Stress and Stay Well. San Francisco: Jossey-Bass (1987).

[24] Flensborg-Madsen T Ventegodt S Merrick J. Sense of coherence and physical health. A review of previous findings. ScientificWorldJournal. (2005) 5:665–73. 10.1100/tsw.2005.85 16127599 PMC5936555

[25] Grødal K Innstrand ST Bauer GF Haugan G Rannestad T André B. Validation of the Norwegian version of the work-related sense of coherence scale. Scand J Public Health. (2018) 46:711–7. 10.1177/1403494817725466 28825350

[26] Grødal K Innstrand ST Haugan G André B. Affective organizational commitment among nursing home employees: a longitudinal study on the influence of a health-promoting work environment. Nurs Open. (2019) 6:1414–23. 10.1002/nop2.338 31660169 PMC6805324

[27] Eriksson M Kerekes N Brink P Pennbrant S Nunstedt H. The level of sense of coherence among Swedish nursing staff. J Adv Nurs. (2019) 75:2766–72. 10.1111/jan.14137 31236952

[28] Del Pozo-Antúnez JJ Ariza-Montes A Fernández-Navarro F Molina-Sánchez H. Effect of a job demand-control-social support model on accounting professionals' health perception. Int J Environ Res Public Health. (2018) 15:2437. 10.3390/ijerph15112437 30388812 PMC6265784

[29] Lecca LI Campagna M Portoghese I Galletta M Mucci N Meloni M. Work related stress, well-being and cardiovascular risk among flight logistic workers: an observational study. Int J Environ Res Public Health. (2018) 15:1952. 10.3390/ijerph15091952 30205457 PMC6164722

[30] Johnson JV. The impact of workplace social support, job demands and work control upon cardiovascular disease in Sweden. ProQuest Inf Learn. (1986).

[31] Johnson JV Hall EM. Job strain, work place social support, and cardiovascular disease: a cross-sectional study of a random sample of the Swedish working population. Am J Public Health. (1988) 78:1336–42. 10.2105/AJPH.78.10.1336 3421392 PMC1349434

[32] Runeson R Lindgren T Wahlstedt K. Sleep problems and psychosocial work environment among Swedish commercial pilots. Am J Ind Med. (2011) 54:545–51. 10.1002/ajim.20943 21351117

[33] Zijlstra FR Sonnentag S. After work is done: Psychological perspectives on recovery from work. Eur J Work Organ Psychol. (2006) 15:129–38. 10.1080/1359432050051385524885178

[34] Cardini BB Freund AM. Recovery from accumulated strain: the role of daily mood and opportunity costs during a vacation. Psychol Health. (2021) 36:913–33. 10.1080/08870446.2020.1809661 32815733

[35] Blank C Gatterer K Leichtfried V Pollhammer D Mair-Raggautz M Duschek S. Short Vacation Improves Stress-Level and Well-Being in German-Speaking Middle-Managers-A randomized controlled trial. Int J Environ Res Public Health. (2018) 15:130. 10.3390/ijerph15010130 29342844 PMC5800229

[36] Lindgren T Andersson K Dammström BG Norbäck D. Ocular, nasal, dermal and general symptoms among commercial airline crews. Int Arch Occup Environ Health. (2002) 75:475–83. 10.1007/s00420-002-0330-8 12172894

[37] Lundberg O Nyström MP A. simplified way of measuring sense of coherence. Eur J Public Health. (1995) 5:56–9. 10.1093/eurpub/5.1.56

[38] Idler EL Angel RJ. Self-rated health and mortality in the NHANES-I epidemiologic follow-up study. Am J Public Health. (1990) 80:446–52. 10.2105/AJPH.80.4.446 2316767 PMC1404567

[39] Gustafsson K Lindfors P Aronsson G Lundberg U. Relationships between self-rating of recovery from work and morning salivary cortisol. J Occup Health. (2008) 50:24–30. 10.1539/joh.50.24 18285641

[40] Akerstedt T. Work schedules and sleep. Experientia. (1984) 40:417–22. 10.1007/BF01952374 6373355

[41] Fu X Lindgren T Guo M Cai GH Lundgren H Norbäck D. Furry pet allergens, fungal DNA and microbial volatile organic compounds (MVOCs) in the commercial aircraft cabin environment. Environ Sci Process Impacts. (2013) 15:1228–34. 10.1039/c3em30928b 23644832

[42] Sun Y Fu X Li Y Yuan Q Ou Z Lindgren T. Shotgun metagenomics of dust microbiome from flight deck and cabin in civil aviation aircraft. Indoor Air. (2020) 30:1199–212. 10.1111/ina.12707 32578244

[43] Sun Y Zhang M Ou Z Meng Y Chen Y Lin R. Indoor microbiome, microbial and plant metabolites, chemical compounds, and asthma symptoms in junior high school students: a multicentre association study in Malaysia. Eur Respir J. (2022) 60:2200260. 10.1183/13993003.00260-2022 35618276 PMC9647074

[44] Fu X Ou Z Zhang M Meng Y Li Y Wen J. Indoor bacterial, fungal and viral species and functional genes in urban and rural schools in Shanxi Province, China-association with asthma, rhinitis and rhinoconjunctivitis in high school students. Microbiome. (2021) 9:138. 10.1186/s40168-021-01091-0 34118964 PMC8199840

[45] Fu X Ou Z Sun Y. Indoor microbiome and allergic diseases: from theoretical advances to prevention strategies. Eco-Environ Health (2022). 10.1016/j.eehl.2022.09.002 PMC10702906 38075599

[46] Gebriné KÉ Lampek K Sárváry A Sárváry A Takács P Zrínyi M. Impact of sense of coherence and work values perception on stress and self-reported health of midwives. Midwifery. (2019) 77:9–15. 10.1016/j.midw.2019.06.006 31233991

[47] Ramberg J Låftman SB Nilbrink J Olsson G Toivanen S. Job strain and sense of coherence: Associations with stress-related outcomes among teachers. Scand J Public Health. (2022) 50:565–74. 10.1177/14034948211011812 33977811 PMC9203657

[48] Wippermann CE Grevenstein MD Nagy E Neubert JC Verres R Kröninger-Jungaberle H. Sense of Coherence und Konsum psychoaktiver Substanzen bei Jugendlichen. Z Kinder Jugendpsychiatr Psychother. (2015) 23:31–42. 10.1026/0943-8149/a000133

[49] Eriksson M Lindström B. Validity of Antonovsky's sense of coherence scale: a systematic review. J Epidemiol Community Health. (2005) 59:460–6. 10.1136/jech.2003.018085 15911640 PMC1757043

[50] Vastamäki J Moser K Paul KI. How stable is sense of coherence? Changes following an intervention for unemployed individuals. Scand J Psychol. (2009) 50:161–71. 10.1111/j.1467-9450.2008.00695.x 18980600

[51] Braun-Lewensohn O. Coping resources and stress reactions among three cultural groups one year after a natural disaster. Clin Soc Work J. (2013) 42: 366–74. 10.1007/s10615-013-0463-0

[52] Weissbecker I Salmon P Studts JL Floyd AR Dedert EA Sephton SE. Mindfulness-based stress reduction and sense of coherence among women with fibromyalgia. J Clin Psychol Med Settings. (2002) 9:297–307. 10.1023/A:1020786917988

[53] Ley C Rato Barrio M Koch A. “In the Sport I Am Here”: Therapeutic Processes and Health Effects of Sport and Exercise on PTSD. Qual Health Res. (2018) 28:491–507. 10.1177/1049732317744533 29199529 PMC5764144

[54] Kekäläinen T Kokko K Sipilä S Walker S. Effects of a 9-month resistance training intervention on quality of life, sense of coherence, and depressive symptoms in older adults: randomized controlled trial. Qual Life Res. (2018) 27:455–65. 10.1007/s11136-017-1733-z 29124498 PMC5846971

[55] Balaj M McNamara CL Eikemo TA Bambra C. The social determinants of inequalities in self-reported health in Europe: findings from the European social survey (2014) special module on the social determinants of health. Eur J Public Health. (2017) 27:107–14. 10.1093/eurpub/ckw217 28355634

[56] Toch-Marquardt M. Does the pattern of occupational class inequalities in self-reported health depend on the choice of survey? A comparative analysis of four surveys and 35 European countries. Eur J Public Health. (2017) 27:34–9. 10.1093/eurpub/ckw228 28355644

[57] Keramat SA Alam K Ahinkorah BO Islam MS Islam MI Hossain MZ. Obesity, Disability and Self-Perceived Health Outcomes in Australian Adults: a Longitudinal Analysis Using 14 Annual Waves of the HILDA Cohort. Clinicoecon Outcomes Res. (2021) 13:777–88. 10.2147/CEOR.S318094 34522108 PMC8434893

[58] Khalaila RN. Socioeconomic status, health behaviors, obesity and self-rated health among older arabs in Israel. J Cross Cult Gerontol. (2017) 32:115–30. 10.1007/s10823-016-9301-5 27484326

[59] Han S. Physical activity and self-rated health: role of contexts. Psychol Health Med. (2021) 26:347–58. 10.1080/13548506.2020.1738016 32151154

[60] Oftedal S Kolt GS Holliday EG Stamatakis E Vandelanotte C Brown WJ. Associations of health-behavior patterns, mental health and self-rated health. Prev Med. (2019) 118:295–303. 10.1016/j.ypmed.2018.11.017 30476503

[61] Hughes A Kumari M. Age modification of the relationship between C-reactive protein and fatigue: findings from Understanding Society (UKHLS). Psychol Med. (2018) 48:1341–9. 10.1017/S0033291717002872 28994356 PMC6088542

