# Supplementary material for: Self-rated health (SRH), recovery from work, fatigue, and insomnia among commercial pilots concerning occupational and non-occupational factors
Source: Front Public Health. 2023 Jan 4;10:1050776. doi: 10.3389/fpubh.2022.1050776 (PMC9846758; doi:10.3389/fpubh.2022.1050776)
Supplement: Supplementary file 1 [file Data_Sheet_1.docx]

Supplementary Material

# Supplementary Table

**Supplementary Table S1.**

| SOC dimension^a^ | Prevalence (%) |
| --- | --- |
| Manageability |  |
| Seldom or never | 2.5 |
| Sometimes | 36.2 |
| Quite often | 48.0 |
| Very often | 13.0 |
| Meaningfulness |  |
| Seldom or never | 4.8 |
| Sometimes | 26.0 |
| Quite often | 51.4 |
| Very often | 17.5 |
| Comprehensibility |  |
| Very or quite often | 3.1 |
| Sometimes | 16.1 |
| Seldom | 64.7 |
| Never | 16.1 |
| Total SOC | Medium (Min, 25^th^,75^th^, Max) |
| SoCtot | 6 (1, 5, 6, 9) |

1. For SOC: There were five scale level for each dimension: never, seldom, sometimes, quite often, and very often. Some groups with a small number were merged to one, including “seldom or never” for manageability and meaningfulness, and “very or quite often” for comprehensibility.

**Table S2. Associations between SRH, recovery after work, fatigue, and insomnia and occupational and life style factors in single factor analysis.^a^**

| Factor | SRH  estimate (95% CI) | *p* value | Recovery  estimate (95% *CI*) | *p* value | Fatigue  OR (95% *CI*) | *p* value | Insomnia  OR (95% *CI*) | *p* value |
| --- | --- | --- | --- | --- | --- | --- | --- | --- |
| Years of employment ^b^ | 1.02 (1.00, 1.05) | 0.08 | 1.02 (1.00, 1.05) | 0.08 | 0.98 (0.96, 1.01) | 0.21 | 0.99 (0.96, 1.02) | 0.65 |
| Part time | 0.94 (0.65, 1.38) | 0.76 | **1.54 (1.06, 2.20)** | **0.021** | 1.17 (0.78, 1.76) | 0.46 | 0.82(0.54,1.25) | 0.36 |
| Co-pilot | 0.76 (0.49, 1.16) | 0.21 | 0.70 (0.46, 1.06) | 0.09 | 1.26 (0.79, 2.02) | 0.87 | 1.54(0.93,2.57) | 0.10 |
| Type of aircraft |  |  |  |  |  |  |  |  |
| B737 | Ref |  | Ref |  | Ref |  | Ref |  |
| MD80serie | 1.25 (0.76, 2.03) | 0.37 | 1.27 (0.79, 2.05) | 0.32 | 0.84 (0.49, 1.44) | 0.52 | **2.08(1.17,3.68)** | **0.012** |
| A330/340 | 1.35 (0.79, 2.27) | 0.27 | 0.79 (0.47, 1.30) | 0.35 | 1.01(0.57, 1.79) | 0.98 | **2.38(1.28,4.46)** | **0.007** |
| Saab 2000 | 1.32 (0.59, 2.94) | 0.49 | 1.22 (0.57, 2.66) | 0.61 | **0.41 (0.17, 0.97)** | **0.041** | 1.02(0.42,2.47) | 0.96 |
| High demand ^c^ | **0.45 (0.34, 0.60)** | **<0.001** | **0.52 (0.40, 0.68)** | **<0.001** | **2.22 (1.63, 3.13)** | **<0.001** | **1.46 (1.08, 2.01)** | **0.018** |
| Low control ^c^ | 0.87 (0.72, 1.10) | 0.25 | **0.66 (0.52, 0.84)** | **0.001** | 1.16 (0.88, 1.52) | 0.26 | 1.00 (0.78, 1.33) | 0.94 |
| Low support ^c^ | **0.50 (0.37, 0.66)** | **<0.001** | **0.38 (0.29, 0.50)** | **<0.001** | **2.00 (1.55, 3.00)** | **<0.001** | **2.00 (1.42, 2.77)** | **<0.001** |
| SOCtot ^c^ | **1.63 (1.40, 1.88)** | **<0.001** | **1.80 (1.62, 2.08)** | **<0.001** | **0.64 (0.54, 0.76)** | **<0.001** | **0.70(0.60, 0.83)** | **<0.001** |
| Manageability | **1.88 (1.40, 2.51)** | **<0.001** | **1.67 (1.27, 2.19)** | **<0.001** | **0.59 (0.43, 0.81)** | **0.001** | **0.62 (0.45, 0.86)** | **0.004** |
| Meaningfulness | **2.44 (1.16, 3.19)** | **<0.001** | **3.11 (2.36, 4.10)** | **<0.001** | **0.50 (0.37, 0.68)** | **<0.001** | **0.59 (0.43, 0.81)** | **0.001** |
| Comprehensibility | **1.68 (1.23, 2.27)** | **0.001** | **2.03 (1.51, 2.73)** | **<0.001** | **0.53 (0.37, 0.76)** | **0.001** | **0.62 (0.43, 0.89)** | **0.010** |
| BMI | **0.39 (0.26, 0.61)** | **<0.001** | 0.73 (0.49, 1.09) | 0.13 | **1.67 (1.06, 2.63)** | **0.026** | 1.24(0.77,2.00) | 0.38 |
| Smoking |  |  |  |  |  |  |  |  |
| Non-smokers | Ref |  | Ref |  | Ref |  | Ref |  |
| Quit | **0.46 (0.28, 0.78)** | **<0.003** | 0.86 (0.53, 1.40) | 0.56 | 1.76 (0.99, 3.12) | 0.05 | 1..49(0.81,2.73) | 0.20 |
| Current smokers | 0.52 (0.21, 1.30) | 0.16 | 0.49 (0.20, 1.16) | 0.10 | 1.09 (0.41, 2.93) | 0.86 | 1.20(0.41,3.47) | 0.74 |
| Habit of using snuff |  |  |  |  |  |  |  |  |
| Non | Ref |  | Ref |  | Ref |  | Ref |  |
| Quit | 0.73 (0.41, 1.30) | 0.28 | 1.06 (0.61, 1.86) | 0.83 | 1.09 (0.59, 205) | 0.78 | 1.22(0.62,2.42) | 0.56 |
| Current use | **0.52 (0.30, 0.89)** | **0.017** | 0.74 (0.44, 1.25) | 0.26 | 1.31 (0.73, 2.36) | 0.37 | 1.15(0.62,2.14) | 0.66 |
| Marital status ^d^ |  |  |  |  |  |  |  |  |
| Married/ couple | Ref |  | Ref |  | Ref |  | Ref |  |
| Weekend Couple | **0.33 (0.12, 0.93)** | **0.037** | **0.31 (0.12, 0.84)** | **0.021** | 1.69(0.52,5.56) | 0.39 | 2.76(0.60,12.64) | 0.19 |
| Single | 0.48 (0.22, 1.05) | 0.07 | 0.50 (0.24, 1.06) | 0.07 | 2.06(0.80,5.31) | 0.14 | 1.79(0.65,4.92) | 0.26 |
| Age of children |  |  |  |  |  |  |  |  |
| None | Ref |  | Ref |  | Ref |  | Ref |  |
| 7-18 | 1.14 (0.68, 1.90) | 0.62 | 0.71 (0.44, 1.16) | 0.18 | 0.84 (0.49, 1.46) | 0.54 | 0.90(0.50-1.59) | 0.70 |
| 0-6 | 0.85 (0.43, 1.68) | 0.64 | **0.42 (0.22, 0.81)** | **0.009** | 0.98 (0.47, 2.03) | 0.95 | 1.98(0.87,4.54) | 0.11 |
| Exercise frequency | **1.80 (1.39, 2.34)** | **<0.001** | **1.39 (1.08, 1.77)** | **0.009** | 0.79 (0.60, 1.05) | 0.11 | 0.82(0.61,1.11) | 0.20 |
| Free hour after work | **1.26 (1.01, 1.58)** | **0.04** | **1.49 (1.20, 1.86)** | **<0.001** | 0.82 (0.64, 1.05) | 0.12 | **0.69(0.53,0.90)** | **0.005** |
| Sleep length |  |  |  |  |  |  |  |  |
| 6-8h | Ref |  | Ref |  | Ref |  | Ref |  |
| 4-5h | **0.09 (0.11, 0.96)** | **0.042** | **0.90 (0.11, 0.96)** | **0.014** | 0.65 (0.30, 1.41) | 0.28 | NA^e^ | NA^e^ |
| >8h | 0.56 (0.27, 1.16) | 0.12 | 0.56 (0.27,1.16) | 0.71 | 3.60 (0.78, 16.62) | 0.10 | 0.51 (0.24, 1.11) | 0.09 |

1. The associations between dependent variables and each factor, except years of employment, were calculated by ordinal regression models, adjusted by age, gender, BMI, smoking habit, and oral tobacco (snuff) use.
2. The associations between dependent variables and years of employment were only adjusted by gender, because of the strong correlation between years of employment and age.
3. The associations between dependent variables and the SOCtot and psychosocial variables were calculated by their interquartile range.
4. Some people are in a stable relationship, and they live together with their partner. They are defined as couple. Some people are in a relationship, but they live separately from their partner, and they usually meet on weekends. They are defined as weekend couple.
5. NA: Not avalabale.
